# Interactions between Kuroshio Extension and Central Tropical Pacific lead to preferred decadal-timescale oscillations in Pacific climate

**DOI:** 10.1038/s41598-019-49927-y

**Published:** 2019-09-19

**Authors:** Youngji Joh, Emanuele Di Lorenzo

**Affiliations:** School of Earth and Atmospheric Sciences, Georgia Institute of Technology, Atlanta, Georgia USA

**Keywords:** Physical oceanography, Physical oceanography

## Abstract

The Kuroshio Extension (KE) exhibits prominent decadal fluctuations that enhance the low-frequency variability of North Pacific climate. Using available observations, we show evidence that a preferred decadal timescale in the KE emerges from the interaction between KE and the central tropical Pacific via Meridional Modes. Specifically, we show that changes in the KE states apply a persistent downstream atmospheric response (e.g. wind stress curl, 0–12 months timescales) that projects on the atmospheric forcing of the Pacific Meridional Modes (PMM) over 9 months timescales. Subsequently, the PMM energizes the central tropical Pacific El Niño Southern Oscillation (CP-ENSO) and its atmospheric teleconnections back to the Northern Hemisphere (1–3 months timescale), which in turn excites oceanic Rossby waves in the central/eastern North Pacific that propagate westward changing the KE (~3 years timescales). Consistent with this hypothesis, the cross-correlation function between the KE and the PMM/CP-ENSO indices exhibits a significant sinusoidal shape corresponding to a preferred spectral power at decadal timescales (~10 years). This dynamics pathway (KE→PMM/CP-ENSO→KE) may provide a new mechanistic basis to explain the preferred decadal-timescale of the North Pacific and enhance decadal predictability of Pacific climate.

## Introduction

The North Pacific climate variability is characterized by strong interactions between ocean and atmosphere on a wide range of timescales. Due to significant influences of the North Pacific decadal fluctuations on marine ecosystems^1–7^ and climate extremes^8–10^, examinations of the mechanism generating a preferred decadal peak in the North Pacific climate spectrum are important. Previous studies have suggested that low-frequency variability of the North Pacific is driven by several mechanisms which include remote forcing from the tropical Pacific^11,12^ and oceanic processes such as anomalous advection acting on the mean thermal gradient^13–15^, the reddening of atmospheric internal variability^16–18^, the reemergence mechanism^19^, and the westward propagation of long Rossby waves^20–23^. Spatially, these mechanisms give rise to a pattern of sea surface temperature (SST) variance that is referred to as the Pacific Decadal Oscillation (PDO)^2^. By decomposing the mechanisms and modes that make up the PDO, Newman *et al*.^24^ reveals that strong quasi-periodic decadal fluctuations of the North Pacific climate are connected to the dynamics of the Kuroshio Extension (KE) region. However, the mechanisms behind a preferred decadal timescale are still being debated.

**Figure 1:**
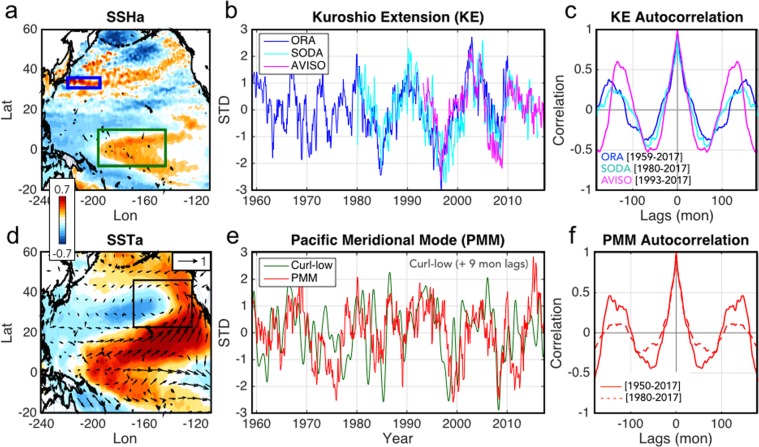
Spatial and temporal structures of the Kuroshio Extension (KE) and Pacific Meridional Modes (PMM) over the historical period. The spatial patterns of the KE and PMM computed by correlating (**a**) SSHa and KE index and (**d**) SSTa/winds and PMM index (see text for definitions). Normalized observational time variations of the (**b**) KE and (**e**) PMM indices between 1959–2017. The green line of (**e**) is the curl downstream index (black line in Fig. 4b) computed by using a downstream curl pattern of black box (Fig. 4a). Autocorrelation functions of (**c**) KE and (**f**) PMM indices with different time periods.

In this study, we combine satellite sea surface height (SSH) data with several reanalysis products, to present observational evidence that a preferred decadal timescale in the KE may arise from the interaction between the KE and the extra-tropical/tropical Pacific variability associated with the Pacific Meridional Modes (PMM) and the El Niño Southern Oscillation (ENSO). Initial support for this hypothesis comes from examining the spatial and temporal structure of the KE SSH anomalies (SSHa) variability (Fig. 1a–c). Specifically, the KE temporal variability, measured by taking a time-series of the average satellite SSHa between 31°–36°N & 140°–165°E (Fig. 1a, blue box, see Data and Methods for details), is characterized by strong low-frequency fluctuations (Fig. 1b) that have a clear decadal spectral peak (~10 years) evident in the auto-correlation function, particularly after the 1976/77 Pacific climate regime shift (Fig. 1c). The spatial footprint of this KE decadal variability extends to the central tropical Pacific variability (Fig. 1a, green box), where several studies have reported a weak, but still significant, preferred decadal spectral peak ~10 years in SST^25–27^ that is identical to that of the KE^21^ (see Supplementary information Fig. 1). These results suggest that the KE and central Pacific interact to give rise to a preferred quasi-decadal period (~10 years), potentially enhancing the decadal predictability of the North Pacific.

## Hypothesis 1: Ocean-Atmosphere Coupling Internal to The North Pacific

**Figure 2:**
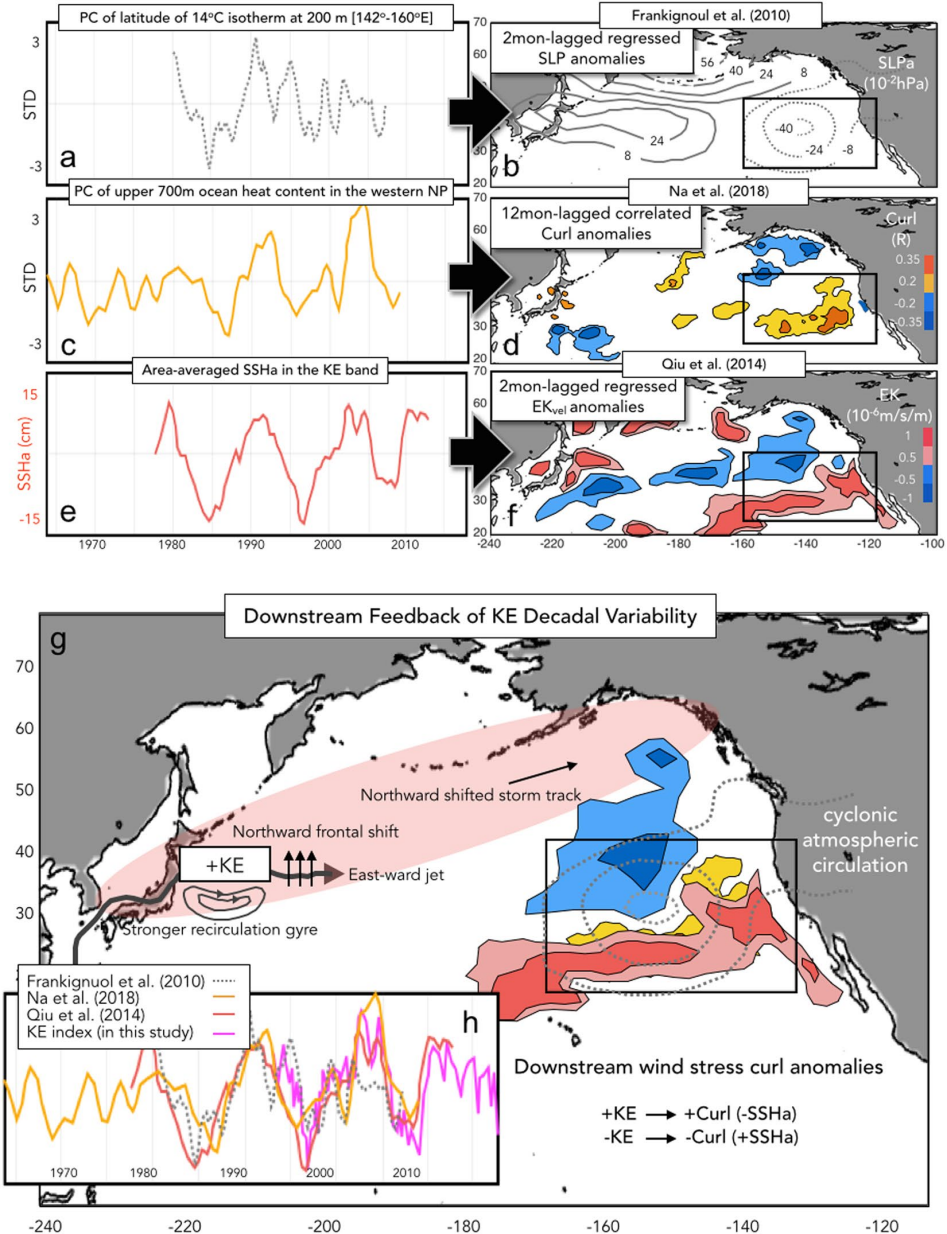
Observational results of the KE feedback from previous studies. The KE indices used in (**a**) Frankignoul *et al*.^36^, (**c**) Na *et al*.^37^, and (**e**) Qiu *et al*.^32^. The corresponding atmospheric/oceanic responses using (**b**) sea level pressure anomalies, (**d**) wind stress curl anomalies, and (**f**) Ekman pumping velocity field anomalies to the positive KE mode (e.g. stable state) with different time lags. (**g**) Illustration of the KE feedback with an integrated KE downstream feedback by -SLP anomalies in (**b**), +Curl anomalies in (**d**) +EK anomalies in (**e**), where the migration of North Pacific storm tracks leads downstream SLP/Curl/Ek responses as described in Qiu *et al*.^32^. (**h**) Comparison of the KE indices from Frankignoul *et al*.^36^ (dashed), Na *et al*.^37^ (yellow), Qiu *et al*.^3^ (red), and this study (pink).

Over the last decade, many studies concluded that the KE region is governed by the North Pacific air-sea coupled system where the decadal changes in the wind forcing over the midlatitude North Pacific are responsible for the transitions of the KE dynamic state (e.g. stable vs. unstable)^17,20,28–32^. Qiu *et al*.^32^ examines the characteristics of the KE decadal system using four dynamic quantities: the length of KE jet, the upstream KE position, the SSH difference across the KE jet, and the KE recirculation gyre strength. For example, through satellite altimeter measurements it is found that the stable state (positive phase) of the KE is characterized by an eastward-extended surface transport of KE jet with a northward migration of latitudinal position of the KE front and an enhancement of the southern recirculation gyre (see schematic Fig. 2g)^32^. The reverse holds when the KE switches to the unstable state (negative phase). Qiu *et al*.^32^ demonstrates that the SSHa variability in the KE southern recirculation box [31°–36°N & 140°–165°E] is an effective way to represent the average fluctuation of those dynamic quantities. The KE SSHa variability between a stable (+SSHa) and an unstable (−SSHa) states (Fig. 1b) are remotely modulated by the arrival of oceanic Rossby waves which are excited by anomalous wind stress curl over the central and eastern North Pacific region^21,23,32,33^. The westward propagating SSHa forcing of KE has been confirmed by subsequent studies with a homvuller diagram of Rossby waves traveling along the midlatitude North Pacific KE band [31°–38°N]^21,23,32,33^.

While the influence of large-scale wind forcing on the KE is well established, the feedback of the KE back to the atmosphere is less understood. Qiu’s^32^ hypothesis states that once the KE is in the stable state, the ocean-to atmosphere heat release and the oceanic front tend to expand northward, leading a northward shift in the North Pacific storm track. This poleward shifted in the storm track is characterized by a dipolar structure of transient eddy temperature flux with a strong north-south gradient along a northeast-southwestward direction. Qiu *et al*. (2014) explains that the dividing line (e.g. maximum gradient) is associated with the region where the time-mean Ekman pumping velocity anomalies field is zero (W_ek_ = 0), which indicates also a northward migration of the W_ek_ response to the KE. The study concludes that the tilted spatial structure of the KE Ekman pumping velocity feedback allows the W_ek_ anomalies in KE band [31°–36°N] to have a sinusoidal spatial pattern, which can excite large-scale oceanic Rossby waves that reach the western boundary back (Fig. 2g)^32,34,35^. Support for this air-sea coupled mechanism of the KE feedback comes from several observational studies that investigate the downstream atmospheric/oceanic response to the KE stable state^32,36,37^ (Fig. 2). Even though the definitions of the KE variability (e.g. indices) differ among these previous studies (Fig. 2a,c,e), a comparison of their time-series reveals strong overlaps in the low-frequency KE variability (Fig. 2h). Similarly, the downstream spatial response found in these studies, despite the use of different response variables like sea level pressure (SLP) anomalies (Fig. 2b), wind stress curl anomalies (Fig. 2d) and Ekman pumping velocity anomaly (Fig. 2f), all show a consistent response (e.g. cyclonic atmospheric circulations) in the subtropical eastern Pacific (Fig. 2g).

**Figure 3:**
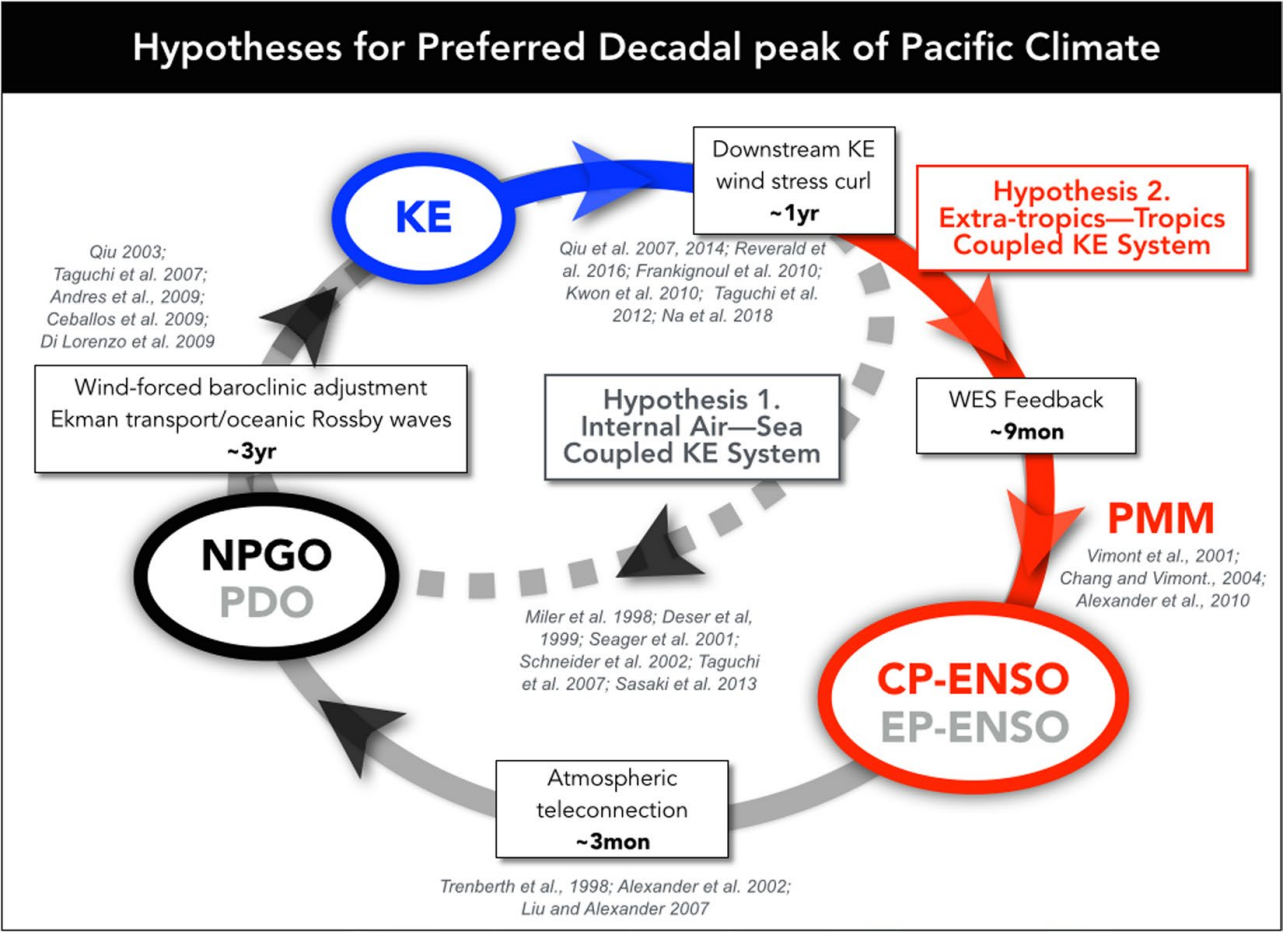
Diagram of hypotheses for generating preferred decadal timescale in Pacific climate variability^64–66^. The internal air-sea coupled KE system proposed by Qiu *et al*.^32^ (KE → KE downstream response → PDO/NPGO → KE, *hypothesis 1*) and the coupled Extra-tropics-tropics KE system suggested in this study (KE → KE downstream response → PMM/CP-ENSO → NPGO → KE, *hypothesis 2*).

In Qiu’s hypothesis, the KE downstream wind forcing (Fig. 2g) is crucial because the generated midlatitude anomalous wind stress curl is thought to project on the same wind forcing patterns that excite the large-scale Rossby waves that propagate into the KE, which would allow the KE system to oscillate over decadal timescale^21,32^. This hypothesis is summarized in a schematic of Fig. 3 (Hypothesis 1). Specifically, the downstream atmospheric response of the KE induces overlying-high and downstream-low SLP anomalies^21,32,36,37^ and may energize the atmospheric variability of the Aleutian Low and/or the North Pacific Oscillation, which in turn drives the two dominant oceanic modes, the PDO and the North Pacific Gyre Oscillation (NPGO) respectively^18^. Once excited, the PDO and NPGO SSHa signals propagate westward into the KE with a ~3 years lag^2,4,24,28,36,38^. This midlatitude wind-SSH coupling of the North Pacific may explain the preferred decadal period ~10 years of the KE and improve the predictive skill of low-frequency KE variability^21,32^. Qiu’s hypothesis states that the preferred time-period for one phase of the KE (~5 years) is obtained by the sum of (1) persistence timescale of the KE index (~1–2 years) and (2) delayed SSH feedback via baroclinic Rossby waves (~3 years).

Although the Qiu’s conceptual model can lead to enhance decadal variance (Fig. 3, Hypothesis 1), a closer examination of the downstream response pattern of the KE shows significant differences from the forcing patterns of the NPGO, PDO and KE in the central and eastern North Pacific. To show this, we begin by defining the spatial and temporal downstream response structure of the KE in the observed wind stress curl anomalies.

### Definition of the KE atmospheric downstream response and Curl Index

**Figure 4:**
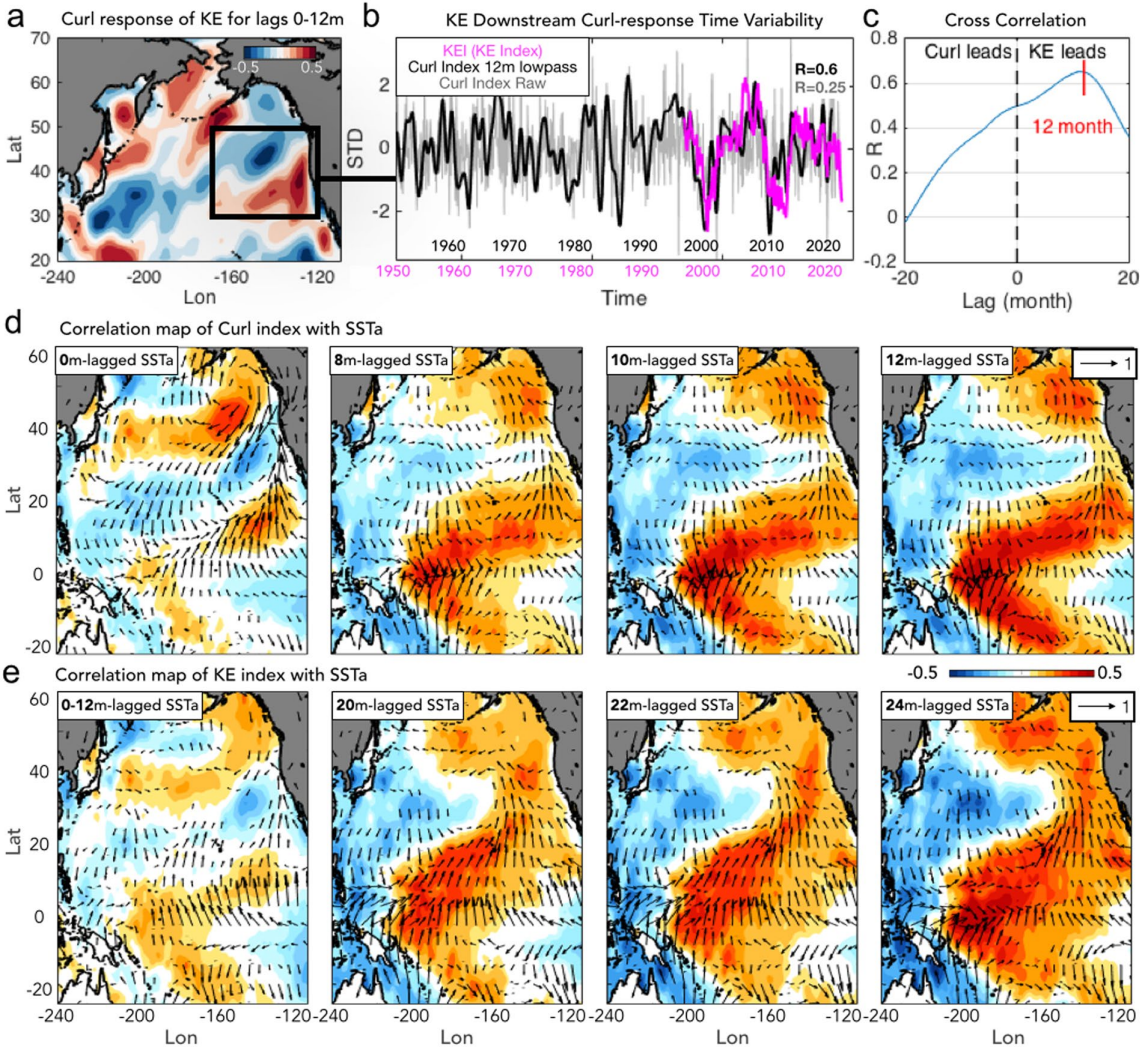
Spatial and temporal structures of downstream wind stress curl feedback of the KE. (**a**) Averaged correlation maps of wind stress curl and the KE index for lag 0–12 months (lagged curl anomalies vs. KE index). (**b**) Comparison between the KE index (pink) and curl indices (12-month lowpass curl index (black) line and raw curl index (gray) lines) with correlation coefficients R (R = 0.6 for the 12-month lowpass curl index, R = 0.25 for the raw curl index both at 99% confidence level). (**c**) Cross-correlation of 12-month lowpass curl index and KE index. (**d**) Correlation maps of 12-month lowpass curl index with lagged SSTa and wind vectors at 0, 8, 10, and 12 month. (**e**) Correlation maps of KE index with lagged SSTa and wind vectors at 0–12 average, 20, 22, and 24 month.

To extract the atmospheric downstream response pattern of the KE (Fig. 4a), we compute a set of correlation maps of the KE index with the wind stress curl for lags 0–12 month and average these maps to obtain the pattern of the KE atmospheric response. These lags are selected based on previous studies that document a persistent downstream atmospheric response on these timescales (see Fig. 2). Prior to the computation of the correlations, a 12 months lowpass filter is applied to the wind stress curl field to better extract the low-frequency response. Given that previous studies have shown that the KE downstream response is persistent from 0–12 months, this averaging approach allows to recover a pattern of response that is consistent with the previous findings (compare Fig. 4a with the patterns from previous studies in Fig. 2). In this framework, the KE downstream response is interpreted as a slow nudging of the storm tracks rather than a sudden change in the atmospheric circulation that is more typical of an ENSO teleconnection (e.g. the excitation of a train of atmospheric Rossby waves). We next use the correlation pattern (Fig. 4a, black box) to define a curl index of the KE downstream response (here and after referred to as the curl downstream index). Specifically, the correlation pattern of Fig. 4a, is projected onto the raw and 12-month lowpass filtered wind stress curl anomalies to extract indices of temporal variability of the wind stress curl pattern in the eastern Pacific region [30^o^–50^o^ N and 170^o^–120^o^ W] outlined by the black box of Fig. 4a. The resulting curl downstream indices (black (12 m lowpass filtered) and gray (raw) lines in Fig. 4b) show significant correlations with the KE index (R = 0.6 with the 12 month lowpass curl index, R = 0.25 with the raw curl index), when the KE index is leading by 12 month. A cross correlation analysis between the KE index and the 12 m lowpass curl index confirms that the strongest correlation is when the KE index leads the curl index by 12 months (Fig. 4c).

### Relation between KE atmospheric response pattern and the NPGO/PDO forcing patterns

**Figure 5:**
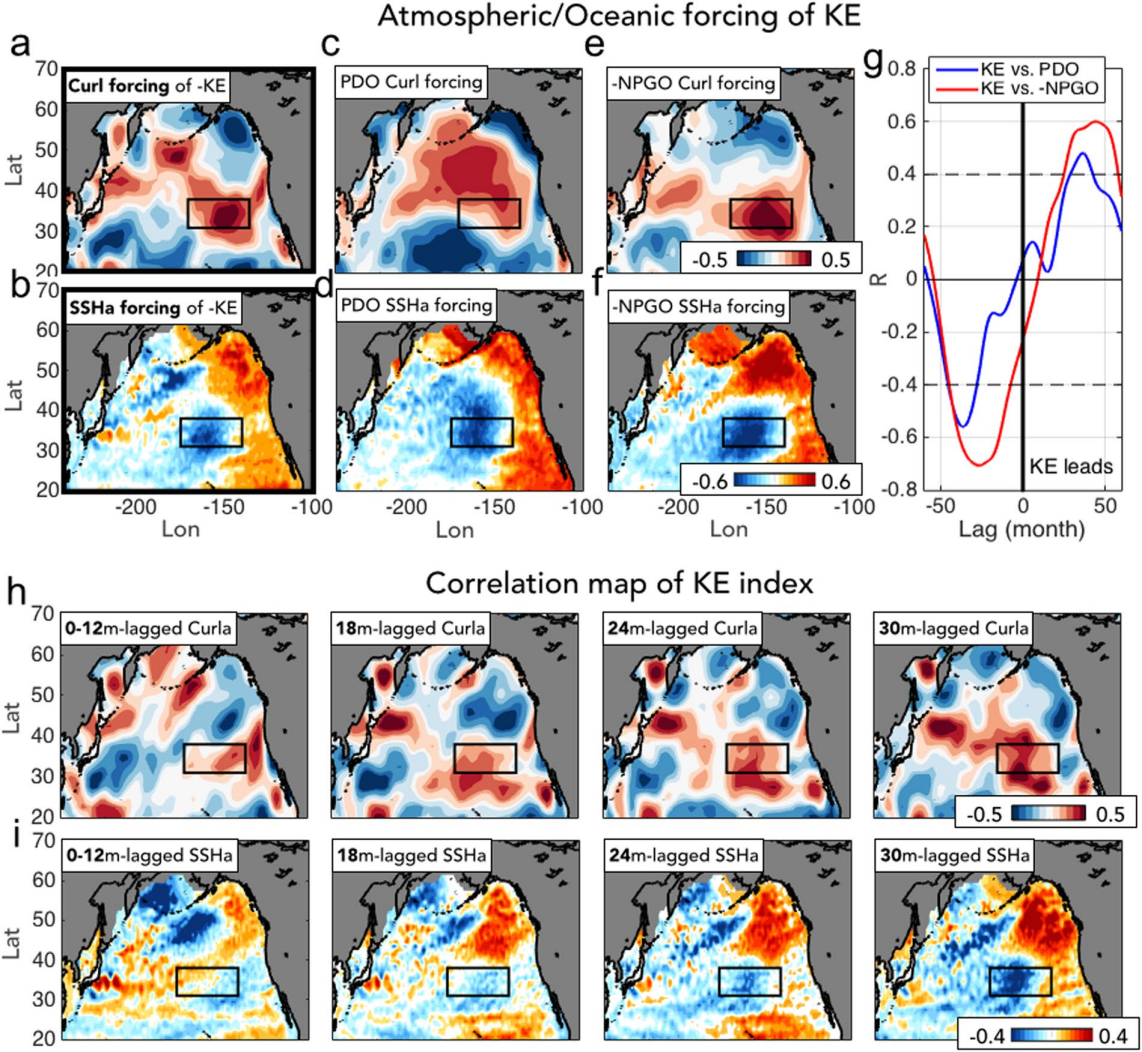
Comparison of KE/PDO/NPGO forcing patterns against the KE downstream response pattern in wind stress curl and SSH field. (**a**,**b**) Correlation maps of -KE index with (**a**) 3yr-leading wind stress curl and (**b**) 3yr-leading SSH anomalies. (**c**,**d**) Correlation maps of PDO index with (**c**) wind stress curl and (**d**) SSH anomalies. (**e**,**f**) Correlation maps of –NPGO index with (**e**) wind stress curl and (**f**) SSH anomalies. (**g**) Cross-correlation of KE and PDO (blue) and KE and -NPGO (red), where the indices are smoothed via 12-month running mean filter with 95% confidence level denoted as dashed lines. (**h**,**i**) Correlation maps of KE index with (**h**) lagged wind stress curl and (**i**) SSH anomalies at 0–12 average, 18, 24, and 30 month.

We now compare the downstream response pattern of the KE (Fig. 4a) with the forcing patterns of the PDO, NPGO and KE in the central and eastern North Pacific (Fig. 5). The forcing patterns for the NPGO and PDO are obtained by correlating the indices of the climate modes with the wind stress curl anomalies (Fig. 5c,e). Similarly, the forcing pattern of the KE is computed by correlating the KE index with the wind stress curl anomalies 36 months earlier to account for the ocean Rossby wave excitation and propagation (Fig. 5a). Consistent with previous studies, we find that the NPGO, and to some extent the PDO, forcing patterns compare well with the spatial structure of the KE forcing pattern (compare Fig. 5a against 5c,e)^20,23^. However, if we compare the KE, NPGO, and PDO forcing patterns (Fig. 5a,c, and e) with the KE downstream response pattern (Fig. 4a), we find important differences and an overall insignificant spatial correlation.

Further inspection of the spatial and temporal structure of the KE atmospheric response in the SSTa and wind stress fields reveals that the KE downstream pattern is more closely related to the forcing pattern of the PMM (see next section and Figs. 4d,e).

### Relation between KE atmospheric downstream response pattern and PMM/CP-ENSO

To examine the KE response pattern in the wind stress curl in more depth, we compute correlation maps of the curl downstream index (Fig. 4b, black line) with SSTa and wind stress anomalies at lags of 0, 8, 10 and 12 months (Fig. 4d). These sets of maps reveal the spatial and temporal forward evolution of the SSTa and winds stress vectors associated with the KE downstream response and are characterized by a progression from a PMM structure (e.g. 0 month lag shows the typical PMM pattern with a weakening of the off-equatorial winds) to a central Pacific El Niño type pattern (lags of 8, 10, 12 months). This suggests that the response pattern of the KE, even though it may have some impact on the forcing patterns of the PDO and NPGO, is more consistent with a PMM/CP-ENSO response. These results lead to a revised hypothesis for explaining the preferred decadal timescale in the KE and central tropical Pacific variability.

## Hypothesis 2: Coupling Between The Extra-Tropical and Tropical Pacific Via Pacific Meridional Modes

The PMM is a coupled SST-surface wind pattern in the mid-latitude North Pacific characterized by the subtropical north-south SST gradient (Fig. 1d) which is driven by the stochastic atmospheric forcing (e.g. North Pacific Oscillation, NPO)^39,40^. We hypothesize that the KE downstream wind stress response, discussed in the previous section (Fig. 4), contributes to the forcing of the PMM, which triggers the air-sea interactions over the Northeast Pacific (e.g. Wind-Evaporation-SST (WES) feedback^41^). The midlatitude Pacific thermodynamic mechanism that relies on ocean-atmosphere coupling could explain the observed persistence to the downstream ocean-atmosphere anomalies, and favor the development of the PMM through weakening the trade winds (Fig. 4d) and the consequent growth of the subtropical Pacific SSTa (KE→PMM, Hypothesis 2 in Fig. 3).

Given that the KE downstream atmospheric response projects onto the excitation region of the PMM and develops into the CP-ENSO type pattern (Fig. 4d), it is highly plausible that the KE can also impact tropical climate through the well-known ENSO precursor dynamics associated with the PMM^42–44^ (PMM/CP-ENSO, Hypothesis 2 in Fig. 3). During the PMM, the WES feedback enables southward coevolution of SSTa and wind stress anomalies that reach the central tropical Pacific where they induce ENSO favorable condition^45^. The excitation, propagation and arrival of the PMM anomalies in the tropics can lead to both flavors of ENSO on timescale of 9 months (see Supplementary Fig. 2). The excited ENSO atmospheric teleconnections (e.g. Pacific/North American pattern) then project the variance back into the central/eastern North Pacific on timescales of 1–3 months^11,12,46^, leading to a deeper than normal Aleutian low (e.g. in the case of an eastern Pacific ENSO) or southern lobe of the NPO (e.g. in the case of a CP-ENSO, see Supplementary Fig. 2). The atmospheric anomalies in the Aleutian Low or NPO excite and drive the westward-propagating oceanic Rossby waves (e.g. the PDO and NPGO SSHa signals) that reach and impact the KE state on timescales of 2.5–3 years^20,22,23,30,47^. This sequence of teleconnections is summarized in Hypothesis 2 in Fig. 3: KE (12 months)→ KE downstream response (9 months) → PMM/CP-ENSO (1–3 months)→ NPGO (36 months)→ KE (*note that while the low-frequency expression of this sequence reveals a CP-ENSO/NPGO signatures*, *on interannual timescales we don’t exclude contributions from eastern Pacific ENSO/PDO*). Consistent with this view, the timescales of the KE/PMM fluctuations are quasi-identical especially since the 1976–77 regime shift (Fig. 1b,e) with the equivalent preferred oscillation period of ~10 years (Fig. 1c,f, power spectra and coherence of the KE and PMM are shown in Supplementary Fig. 1).

Using hypothesis 2 in Fig. 3 as a roadmap for the proposed KE dynamic pathway, we explore the observational evidence associated with the dynamics pathway linking the KE to the central tropical Pacific. Specifically, we examine more closely the role of the KE downstream atmospheric response in the eastern Pacific as forcing of the PMM and central tropical Pacific variability. The link between ENSO teleconnections and the North Pacific modes (see Supplementary Fig. 3) will not be discussed given the large amount of existing literature documenting this part of the loop in hypothesis 2 (Fig. 3). However, we will examine more closely the patterns of wind stress curl associated with the modes in relation to the atmospheric forcing pattern of the KE.

### Impact of KE downstream atmospheric response on PMM forcing in eastern Pacific

Support for Hypothesis 2 (KE→PMM in Hypothesis 2 of Fig. 3) comes from exploring the spatial evolution of the KE downstream atmospheric response in the ocean and atmosphere obtained by correlating the KE and curl downstream index with wind and SST anomalies at different lags in time (Fig. 4d,e). The spatial progression of the curl downstream associated with the KE index shows a persistent cyclonic circulation over the Northeast Pacific, which projects onto a weakening of the off-equatorial trade winds and leads to the growth of subtropical warm SSTa through the WES feedback^45^. As the subtropical SSTa continue to grow, the PMM-like oceanic signature becomes more evident with strong wind vector anomalies that transition into the mature phase of the PMM (e.g. compare Fig. 4e at lag 24 month with Fig. 1d).

The forcing of PMM associated with the KE atmospheric downstream feedback is also captured in a comparison of the time-series of the PMM and the curl downstream index (Fig. 1e). Significant correlation is found when the curl downstream leads the PMM by 9 month with R = 0.39 over the entire period and R = 0.54 after 1976 at 95% and 99% significance level respectively.

### The atmospheric forcing of KE and its relation to the North Pacific modes

The link between the tropical variability (e.g. ENSO) and the North Pacific decadal modes via the “atmospheric bridge” is well established by previous literature^11,12,46^. Once the ENSO atmospheric teleconnections project the tropical signal into the North Pacific, changes in the surface heat fluxes, wind-driven mixing, and Ekman pumping in the upper ocean drive the PDO-like and NPGO-like SSH signals that affect the KE system through westward propagating Rossby waves. Consistent with the previous findings, we find that the PDO/NPGO curl/SSH patterns show a significant link of the KE atmospheric forcing (Fig. 5a,c,e) and its SSH signature (Fig. 5b,d,f) over the eastern North Pacific region. The cross correlation of the KE and the PDO/NPGO indices confirms that the KE has been interacting with the North Pacific decadal modes on quasi-decadal time scale, with significant lead/lag correlations especially to the NPGO (R > 0.6, Fig. 5g).

As we already noted in the previous section, the spatial progression of the KE index in wind stress curl and SSH field reveals that the KE atmospheric/oceanic downstream response patterns (Fig. 5h,i at lag 0–12 month) are not correlated to the NPGO forcing patterns, showing a significant structural difference in the eastern North Pacific region (e.g. Fig. 5h at lag 0–12 month vs. Fig. 5e). Specifically, from the lagged 18-month, the tilted dipole structure of KE downstream wind stress curl changes into a blob pattern and increases in amplitude, as observed in the KE wind forcing pattern over the subtropical Northeast Pacific (Fig. 5h). Corresponding to the spatial progression of KE curl feedback, the SSH progression also reveals the delayed response, showing that the significant eastern North Pacific SSHa appear only after the lagged 18-month (compare Fig. 5i with Fig. 5b).

Taken together, the examination of KE atmospheric/oceanic downstream response at lag 0-12 month (Fig. 5h,i) and forcing patterns (Fig. 5a,b) implies that the KE downstream feedback might not be the decisive forcing to excite the baroclinic Rossby waves that effectively switch the KE phase. Instead, the eastern North Pacific wind forcing energized by the atmospheric teleconnections associated to the PMM/CP-ENSO (see Fig. 4d,e) plays a more important role in modulating the decadal KE dynamic state by projecting onto the PDO and NPGO SSH anomalies that lead the phase transitions in the KE.

### 10 year spectral peak in the co-evolution of KE and PMM/CP-ENSO

**Figure 6:**
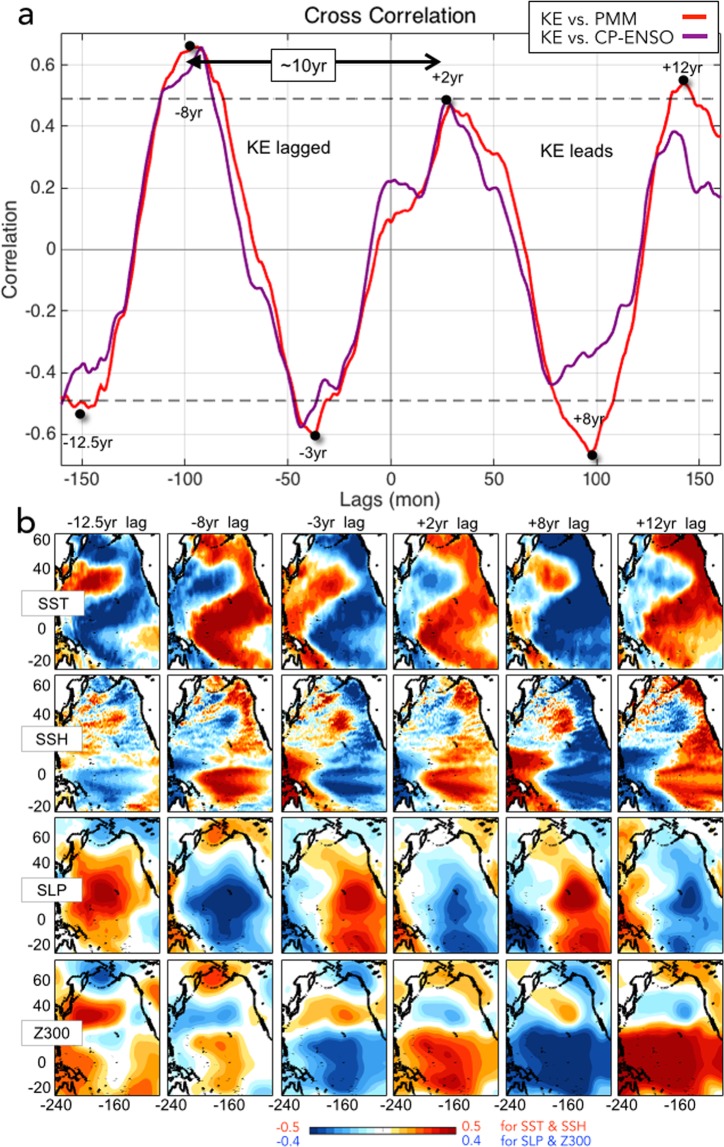
Temporal and spatial co-evolutions of KE and PMM/CP-ENSO. (**a**) Cross-correlation functions between the KE index with PMM and CP-ENSO indices. (**b**) Correlation maps between the KE index and oceanic/atmospheric variables (b-row1: SST, b-row2: SSH, b-row3: SLP, and b-row4: 300hPa geopotential height, Z300) using different time lags.

The dynamical pathway of hypothesis 2 (Fig. 3) suggests that the one phase of decadal KE state persists ~5 years with ~10 years oscillation timescales following the low-frequency sequence of KE (12 months)→ KE downstream response (9 months) → PMM/CP-ENSO (1–3 months)→ NPGO (36 months)→ KE. This progression is evident from the temporal and spatial lead/lag relationships between the KE and PMM/CP-ENSO indices (Fig. 6). As for the CP-ENSO index, the C index of Takahashi *et al*.^48^ was utilized by employing two principal components of SSTa in the tropical Pacific, whose spatial signature is only constrained to the central Pacific anomalies. Using this definition of the CP-ENSO and the PMM index, we note that the CP-ENSO decadal fluctuations are significantly correlated with the PMM at lag 0 (Supplementary Fig. 4), which is consistent with the recent findings that the CP-ENSO is not only driven by the PMM but also interacts with the PMM on interannual timescales through the fast-positive feedback^49^. We find that the cross-correlation functions between the KE and PMM and KE and CP-ENSO (Fig. 6a) both exhibit a clear sinusoidal shape with a preferred spectral power at the decadal timescale of ~10 years, which is particularly strong since the 1976/77 Pacific climate regime shift. The lead-lag relationship between the KE and the PMM is very significant with average positive and negative correlations above the 95% confidence level. The significant sinusoidal shape of the cross-correlations reveals that the KE and PMM/CP-ENSO have been interacting with each other on decadal timescales.

To inspect the spatial patterns that correspond to the temporal cross-correlation function between KE/PMM indices, we compute spatial correlation maps between the KE index and oceanic/atmospheric variables over the period between 1990 to 2017 (Fig. 6b) using different lags associated with the peaks of the cross-correlation function of Fig. 6a. The spatial structures and temporal evolutions of SST (Fig. 6b-row 1), SSH (Fig. 6b-row 2), SLP (Fig. 6b-row 3), and 300 hPa geopotential height (Fig. 6b-row 4) patterns reveal that the decadal KE variability captures the oceanic/atmospheric signatures of the PMM and its progression. We find that the KE index can reconstruct clear transitions between the positive and negative phase of recurring PMM expressions which exhibit the hybrid of PDO-like and NPGO-like signatures with the central Pacific ENSO-like signal (Fig. 6b-row1). The KE-induced SSH patterns (Fig. 6b-row2) reveal that the KE variability is linked to the development of ENSO conditions, including the zonal SSH gradient in the tropical Pacific that generates the equatorial oceanic wave dynamics (e.g. downwelling equatorial Kelvin waves in positive ENSO). Corresponding to the tropical convective system caused by the central Pacific SST warming (Fig. 6b-row1), the lower and upper atmospheric layers exhibit the large-scale east-west SLP seesaw pattern (Fig. 6b-row3) with the characteristic pattern of the atmospheric Rossby wave train to the North Pacific at the 300 hPa (Fig. 6b-row4). These tropical teleconnections to the North Pacific are well documented and are an important source of the reddening of the Pacific climate spectrum^11,12,46,50–53^. Taken together, the temporal interactions between the KE and PMM/CP-ENSO (Fig. 6a) along with the spatial signatures inferred from lead/lag correlations with the KE index (Fig. 6b) support our hypothesis that decadal fluctuations of KE can emerge through a two-way climate coupling between the North Pacific and the tropics.

## Summary and Discussion

Using available observational dataset and reanalyses of the Pacific Ocean, this study explores an additional pathway for generating quasi-decadal fluctuations in the North and central tropical Pacific that relies on a two-way interaction between the KE in the extra-tropical and tropical Pacific. Through statistical analyses of the lead/lag relationship of the KE with large-scale ocean-atmosphere reanalysis, we offer initial evidence that the decadal SSH variability in the KE region is not independent of the PMM/ENSO, especially over the recent decades. The KE-PMM decadal interaction involves three key processes that are associated with the sequence of teleconnections outlined in the Fig. 3 schematic: KE (12 months)→ KE downstream response (9 months) → PMM/CP-ENSO (1–3 months)→ NPGO (36 months)→ KE.

1. The persistent KE downstream atmospheric response (e.g. wind stress curl) projects on the forcing pattern that energizes the PMM and the central Pacific SST warming leading to ENSO conditions.
2. The activation of the ENSO system (Fig. 4d,e)^42–44^ and its atmospheric teleconnections^11,12,39^, contribute to energizing the North Pacific modes, specifically the PDO and NPGO.
3. The PDO and NPGO SSH anomalies propagate westward as Rossby waves impacting the KE state again^20,22,23,30,47^.

Although it is not possible to prove this oscillatory model without an appropriate modeling framework, a detail analysis of the KE downstream response (Fig. 4) and forcing (Fig. 5) patterns is consistent with our revised hypothesis 2. Specifically, we find that the spatial and temporal evolution of the KE atmospheric downstream response is more consistent with the development of a PMM/CP-ENSO expression (Fig. 4) than with the forcing pattern of the KE reverse phase (Fig. 5a). This finding is further supported by the cross-correlation functions between indices of the KE and PMM/CP-ENSO (Fig. 6a), which exhibit a sinusoidal shape with significant ± correlation peaks with ~5 yr intervals. This is indicative of a preferred decadal oscillation of ~10 years (Fig. 6a) and is consistent with the timescale of hypothesis 2: KE (12 months)→ KE downstream response (9 months) → PMM/CP-ENSO (1–3 months)→ NPGO (36 months)→ KE, which is also evident in the spatial progression of oceanic/atmospheric anomalies associated with this cross-correlation function (Fig. 6b). To quantify how the KE signal propagates through the sequence we examined the signal to noise (SNR = 1/[1 − R^2^]) ratio of the KE index against indices of the wind stress curl, PMM, CP-ENSO, NPGO, and KE using optimal lag correlations (R) at 12, 28, 30, 34, 60 months, which are consistent with the timescale of propagation of the sequence. On average the SNR remains constant at about 1.5 (Supplementary information Fig. 5) suggesting a steady propagation of the signal across the sequence with little loss of variance. The SNR values become higher (~2) if we apply a low-frequency filter (5-year), suggesting that this sequence explains an important fraction of the low-frequency variance of the North Pacific system. As noted earlier, while the low-frequency expression of this sequence reveals CP-ENSO/NPGO signatures, on interannual timescales we don’t exclude contributions from eastern Pacific ENSO/PDO. Further, the low-frequency expression of this sequence may serve as a physical mechanism to a new mode of quasi-decadal climate variability over the North Pacific—the Pacific Decadal Precession (PDP)^54,55^ which is characterized by a ~10 year counter-clockwise progression of an atmospheric pressure dipole around the North Pacific, one phase of which maps onto the NPO and is coincident with CP-ENSO variability discussed here, and another phase of which maps onto (and is coincident with) KE pattern of the atmospheric variability seen in Fig. 2 (e.g. the spatial expression of the KE index).

In this study, the persistence of the KE downstream atmospheric feedback (~1 yr) shown in our results supports the previous view, where the SSH-based KE variability has a long memory and can represent prolonged forcing of ocean dynamics (e.g. modulations of oceanic recirculation gyre and stability of the KE jet) to the North Pacific atmosphere^20,21,23,32,36,37^. Teleconnections excited by the central tropical Pacific SSTa are also known to influence the extra-tropical atmosphere in the region of the KE downstream wind stress curl response pattern^50^. However, the spatial imprint of CP-ENSO onto the curl response pattern of the North Pacific (Fig. S6b) is characterized by a meridional wave train that resembles more closely the North Pacific Oscillation (NPO) and is structurally different from the curl response pattern of the KE (Supplementary information Fig. 6a). Nevertheless, we cannot exclude that the central Pacific SSTa have some impact on, and explain some of the variance (R = 0.1–0.2) of, the KE wind stress curl response pattern, as evident from correlating the index of the curl response pattern with concurrent and leading SSTa in the tropical Pacific (Supplementary information Fig. 6c). This is consistent with some studies suggesting that the CP-ENSO can influence the initiations of the North Pacific PMM in the following season by altering the NPO variability^52^.

Although the analyses presented use monthly data, seasonality plays a key role in the extra-tropical/tropical interactions between the PMM and the ENSO system. While the KE variability is characterized by low-frequency fluctuations that have persistence on timescales of one year (e.g. KE auto-decorrelation length scale ~12 months), the wind stress curl downstream response pattern to KE forcing (Fig. 4a) exhibits a seasonal modulation with maximum amplitude during the Winter (from January to March) (see Supplementary information Fig. 7). This seasonal timing is important because the dynamics of the PMM as precursors to ENSO are also seasonally locked with an initiation in Winter^41,42^, development during Spring, and peak in Summer with the growing ENSO.

The observational evidence in this study provides a mechanistic hypothesis for exploring a new set of decadal climate interactions between the North Pacific western boundary current and the tropical Pacific, which could explain the quasi-preferred decadal peak in the observed spectrum of Pacific climate (e.g. central tropical Pacific, KE system). While we are unable to provide ultimate proof that this interaction is real, given the short observational record, the consistency of observations with this hypothesis provide a road map for designing advanced modeling experiment that can effectively test the relation between the KE and the tropical Pacific. Understanding this interaction becomes even more critical because of recent findings suggesting that the coupling between extra-tropics and tropics is intensifying in a warmer climate^9,10,32,56,57^. The enhanced coupling, which has been implicated in the rising of the Pacific decadal variance, is also consistent with the stronger periodic cross-correlation between the KE and PMM, suggesting that the interaction of KE and PMM may also be changing in future climate. A higher amplitude quasi-decadal fluctuations of the sequence KE/PMM, may lead to a stronger basis for making decadal predictions, especially for societally relevant biogeochemical quantities (e.g., salinity, oxygen, chlorophyll-A) and fisheries that are linked to the KE decadal variability^58^.

## Data and Methods

Observations for investigating the SSH-based KE variability are obtained by three different SSH dataset within the period between 1959 and 2017 from the European Centre for Medium-Range Weather Forecasts (ECMWF) Ocean Reanalysis System: ORA-S3^59^, Simple Ocean Data Assimilation (SODA) reanalysis version 3^60^, and satellite distributed by Archiving, Validation, and Interpretation of Satellite Oceanographic (AVISO) data available at https://www.aviso.altimetry.fr/en/data/products/auxiliary-products/mss.html^61^. To explore the KE variability at the air–sea interface, the monthly mean Hadley Centre Sea Ice and Sea Surface Temperature (HadiSST) dataset^62^ are used, and sea Level pressure (SLP), geopotential height at 300 hPa (Z300), zonal and meridional wind stress are taken from European Centre for Medium-Range Weather Forecasts (ERA) reanalysis product^63^. All anomalies are constructed by removing the mean monthly climatology and linear trend at each grid point. The limited period between 1959 and 2017 is analyzed.

The KE index is defined as monthly area-averaged SSH anomalies in the 31°–36°N & 140°–165°E region (Box in Fig. 1a) following the Qiu *et al*.^32^. The SST-based monthly PMM index was obtained at https://www.esrl.noaa.gov/psd/data/timeseries/monthly/PMM/^42^. The Central Pacific ENSO (CP-ENSO) is defined by utilizing the C-index computed by the sum of the first and the second principal component of tropical SST (Takahashi *et al*.)^48^. All the indices are normalized and detrended before analysis. To show the oceanic/atmospheric spatial responses to the time-varying KE dynamic state and their oscillation patterns, we use lead/lag correlation maps between oceanic/atmospheric anomalies and KE index.

## Supplementary information

Supplementary information
