# Supplementary material for: Interactions between Kuroshio Extension and Central Tropical Pacific lead to preferred decadal-timescale oscillations in Pacific climate
Source: Sci Rep. 2019 Sep 19;9:13558. doi: 10.1038/s41598-019-49927-y (PMC6753113; doi:10.1038/s41598-019-49927-y)
Supplement: Supplementary file 1 — Supplementary information [file 41598_2019_49927_MOESM1_ESM.pdf]

Supplementary information for

Interactions between Kuroshio Extension and Central Tropical Pacific lead to preferred decadal-timescale oscillations in Pacific climate

Youngji Joh[1*] and Emanuele Di Lorenzo[1]

[1]School of Earth and Atmospheric Sciences, Georgia Institute of Technology, Atlanta, Georgia, USA

youngji.joh@gmail.com

Contents of this file

Figures S1-S7.

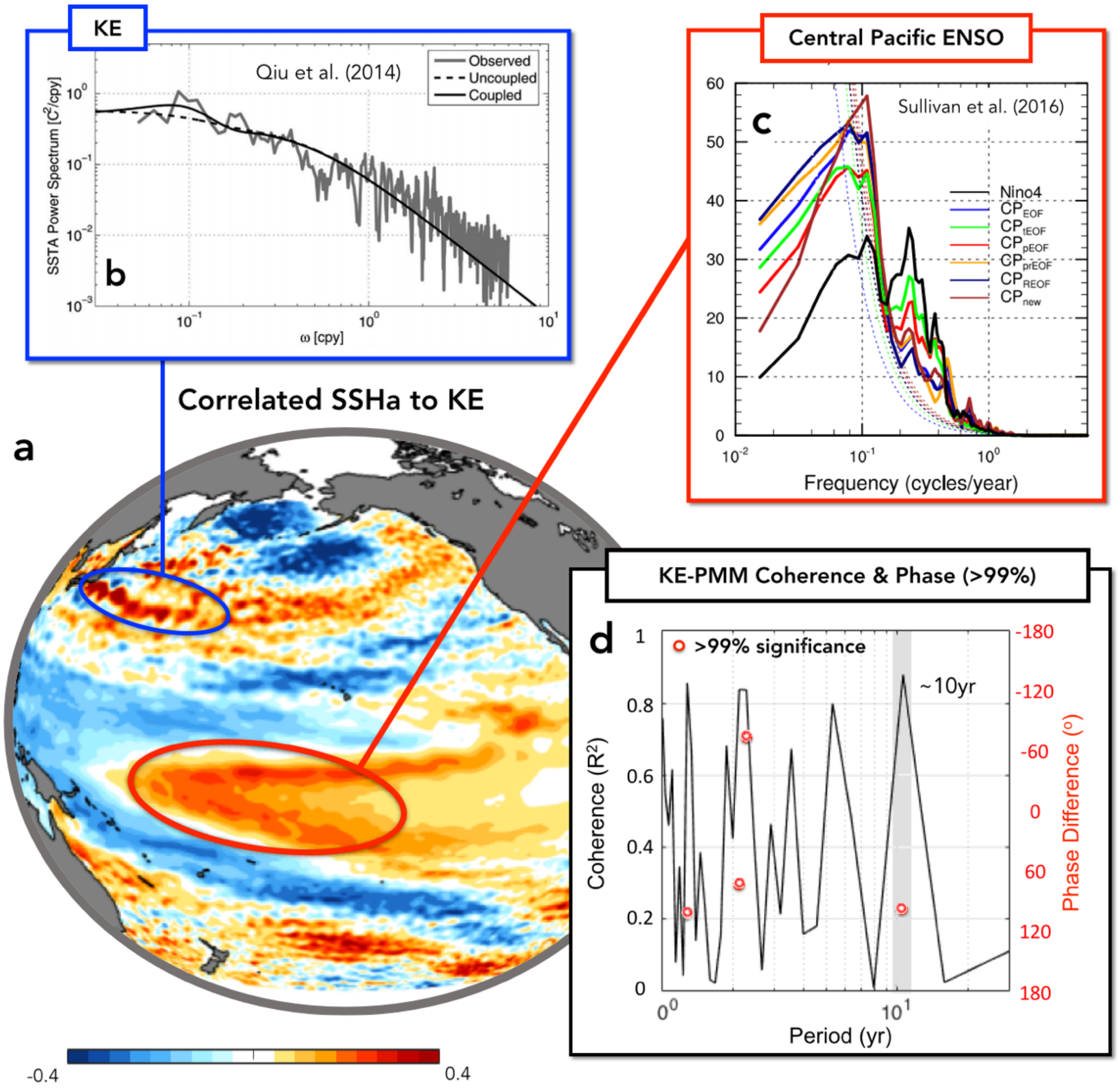

Figure S1. Linkages between the Kuroshio Extension (KE) region and the central tropical Pacific basin with the same preferred decadal-timescale of 10 years. (a) Spatial pattern computed by correlating SSH anomalies with the KE index between 1990-2017 using AVISO data (see text for definitions). (b) Power spectrum of the observed sea surface temperature (SST) anomalies in the KE band (gray line) documented by Qiu et al. (2014)[32], where the power spectrum of SST under the air-sea uncoupled scenario is denoted as dashed line and that under the air-sea coupled scenario as the solid line. (c) Power spectrum of indices (computed following previous studies) of central Pacific El Niño Southern Oscillation (ENSO) documented by Sullivan et al. (2016)[27]. (d) Coherence and phase difference between KE and PMM indices used in this study.

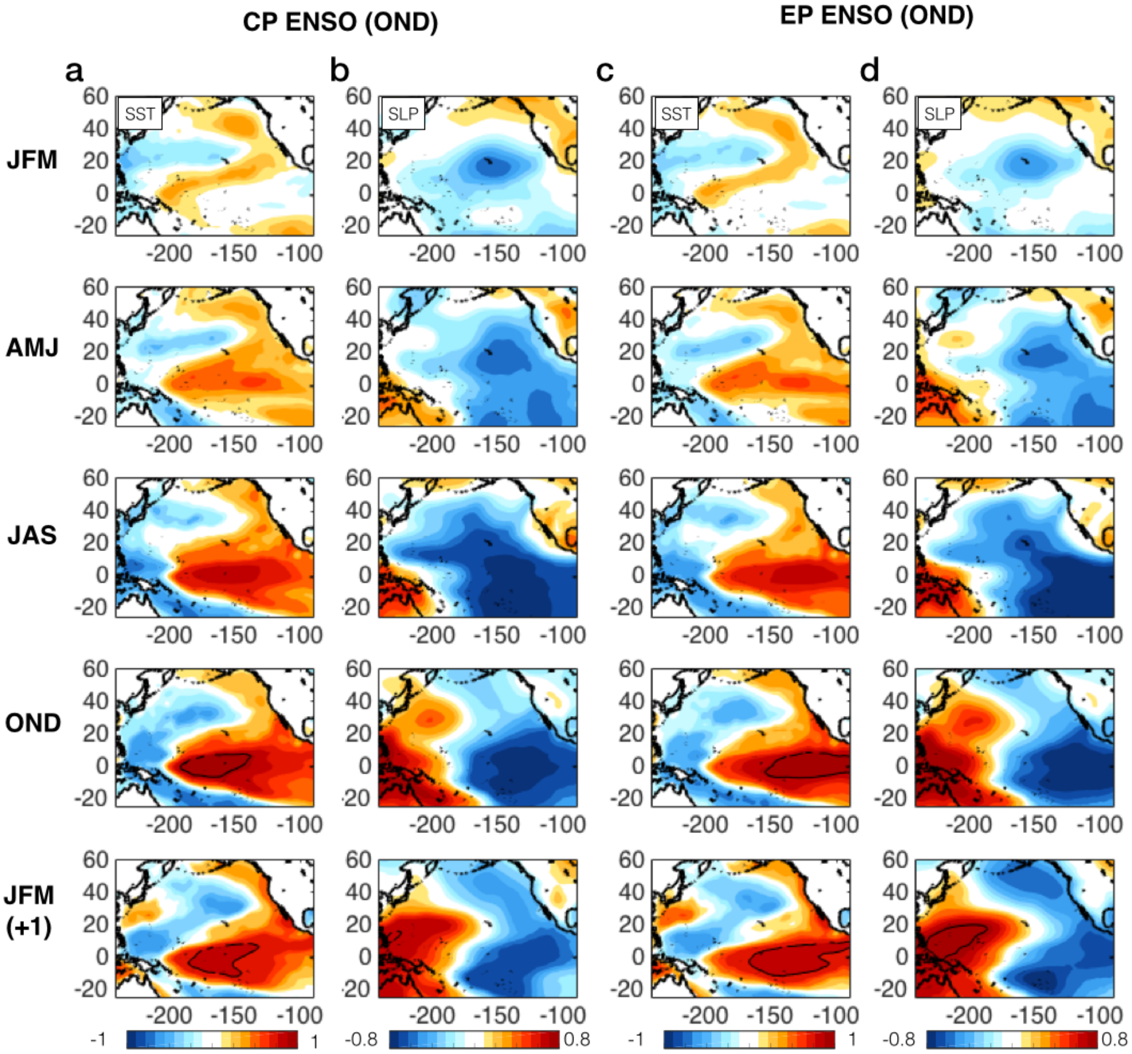

Figure S2. Spatial structure and temporal evolution of CP/EP ENSO in SST and SLP field in observations. (a-b) Correlation between the winter (OND) CP-ENSO and prior JFM, AMJ, JAS, concurrent OND, and following JFM seasonal mean (a) SSTa and (b) SLPa. (c-d) Correlation between the winter (OND) EP-ENSO and prior JFM, AMJ, JAS, concurrent OND, and following JFM seasonal mean (c) SSTa and (d) SLPa. CP/EP ENSO indices were simply defined by the Nino4 and Nino3 indices.

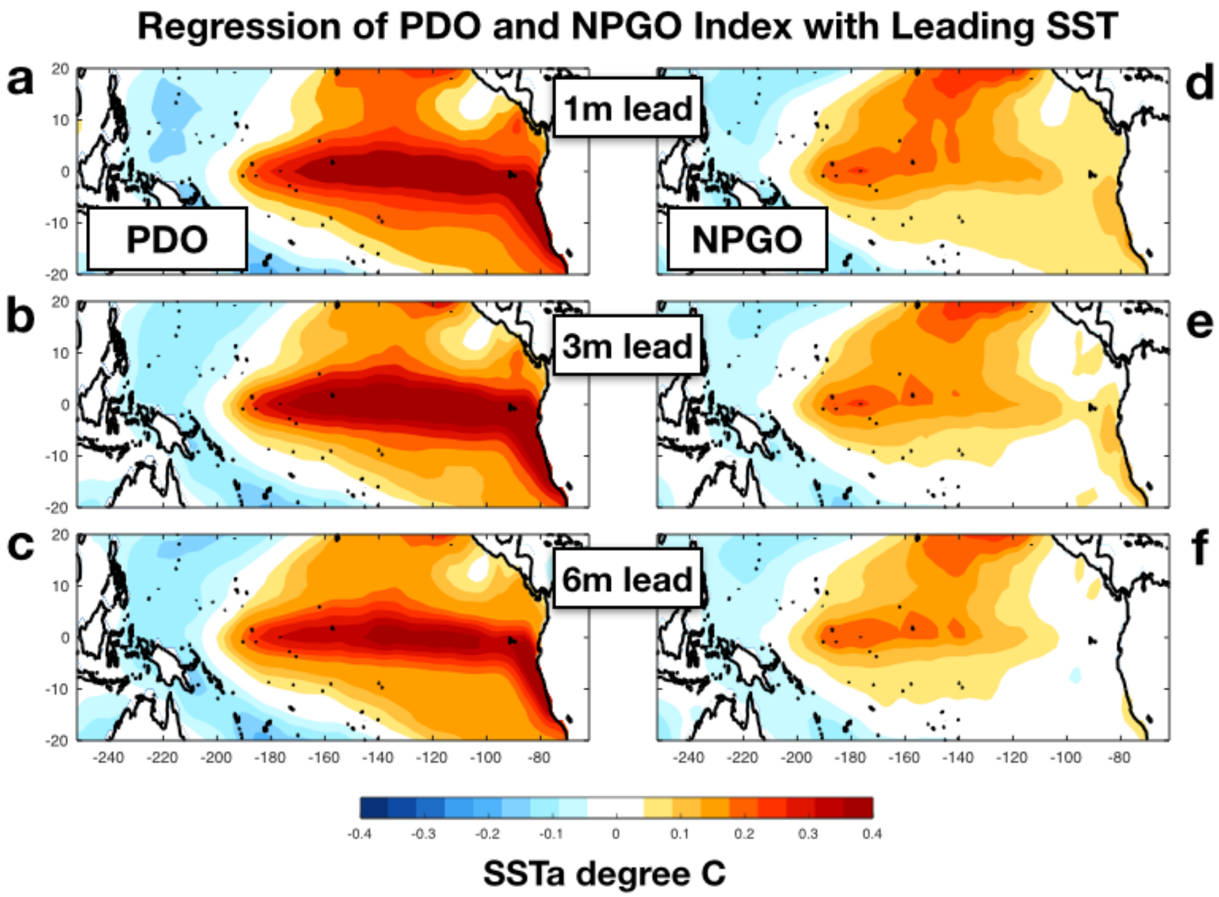

Figure S3. Leading SST patterns to PDO and NPGO. (a-c) Regression maps between the PDO index to leading SSTa by (a) 1 month, (b) 3 month, (c) 6 month. (d-f) Regression maps between the NPGO index to leading SSTa by (a) 1 month, (b) 3 month, (c) 6 month.

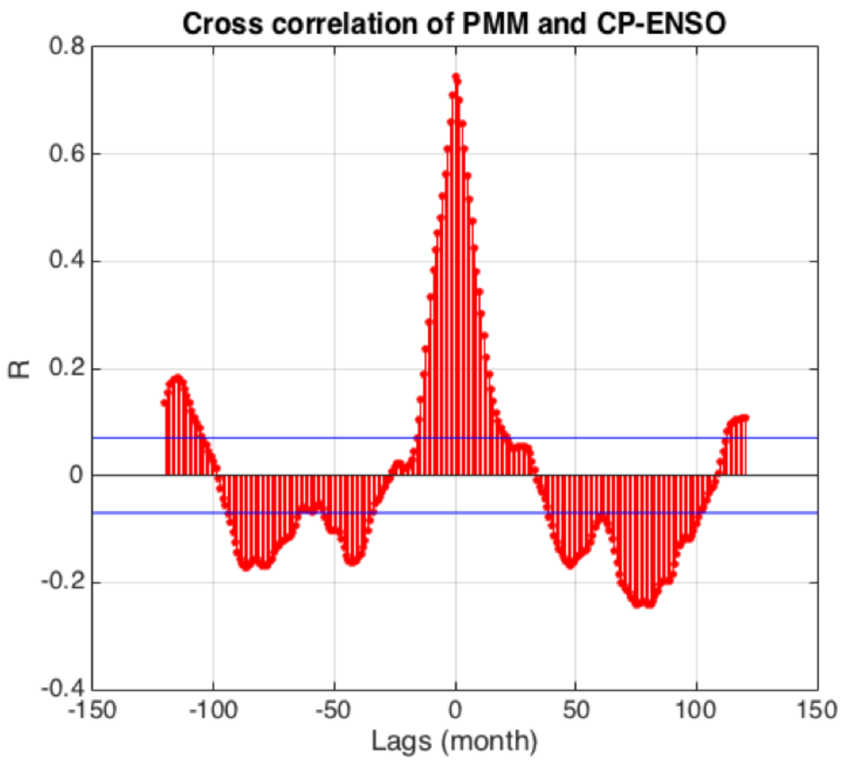

Figure S4. Cross correlation function between the PMM and CP-ENSO indices. The CP-ENSO index was utilized by the C index in Takahashi et al. (2011).

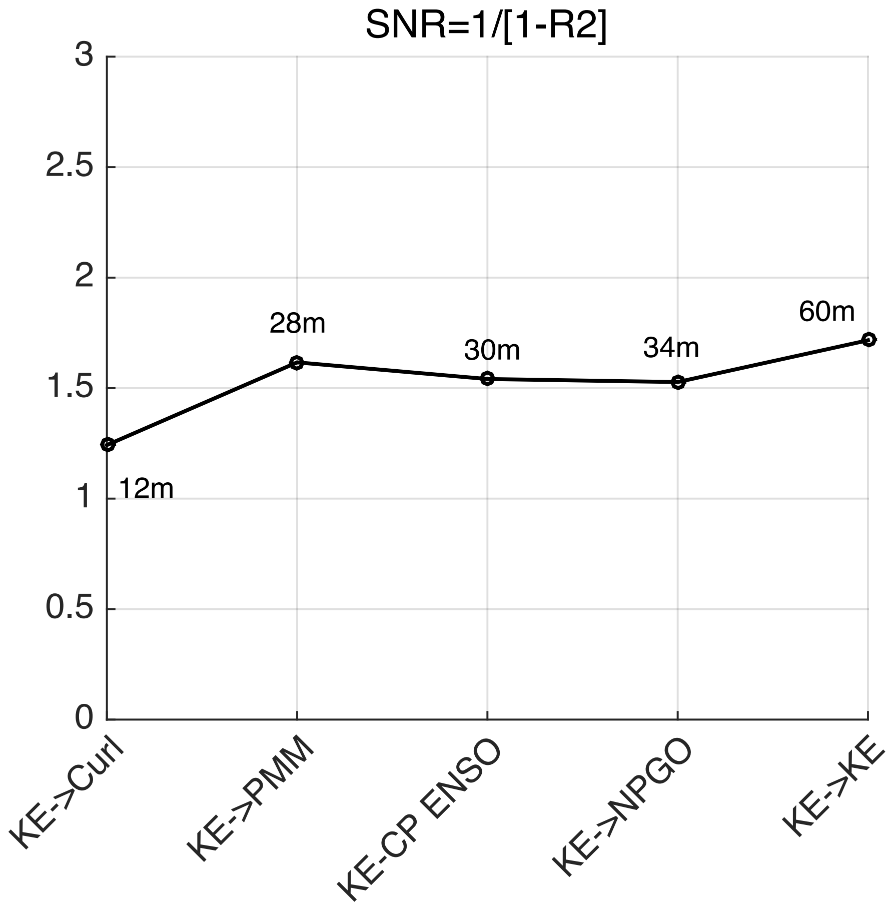

Figure S5. The signal to noise ratio (SNR) of the KE index against indices of KE downstream wind stress curl (Figure 1b), PMM, CP-ENSO, NPGO and KE with lag at 12, 28, 30, 34, and 60 months respectively. The ratio of signal and noise for each step is computed by using a linear regression y = a*x + n model to estimate the explained variance, where x is the KE index, y is a signal, and n is noise. The SNR was considered as signal/noise = var(y)/var(n) $= < y'^2 > / < n'^2 > = 1 / [1 - r(x,y)^2]$ using a relationship between a regression coefficient **a** and a correlation coefficient **r** of x and y in our hindcast model, where n'=y' - a*x; $a = < x'y' > / < x'^2 >$; $r = < x'y' > / (\sqrt{< x'^2 >}\sqrt{< y'^2 >})$.

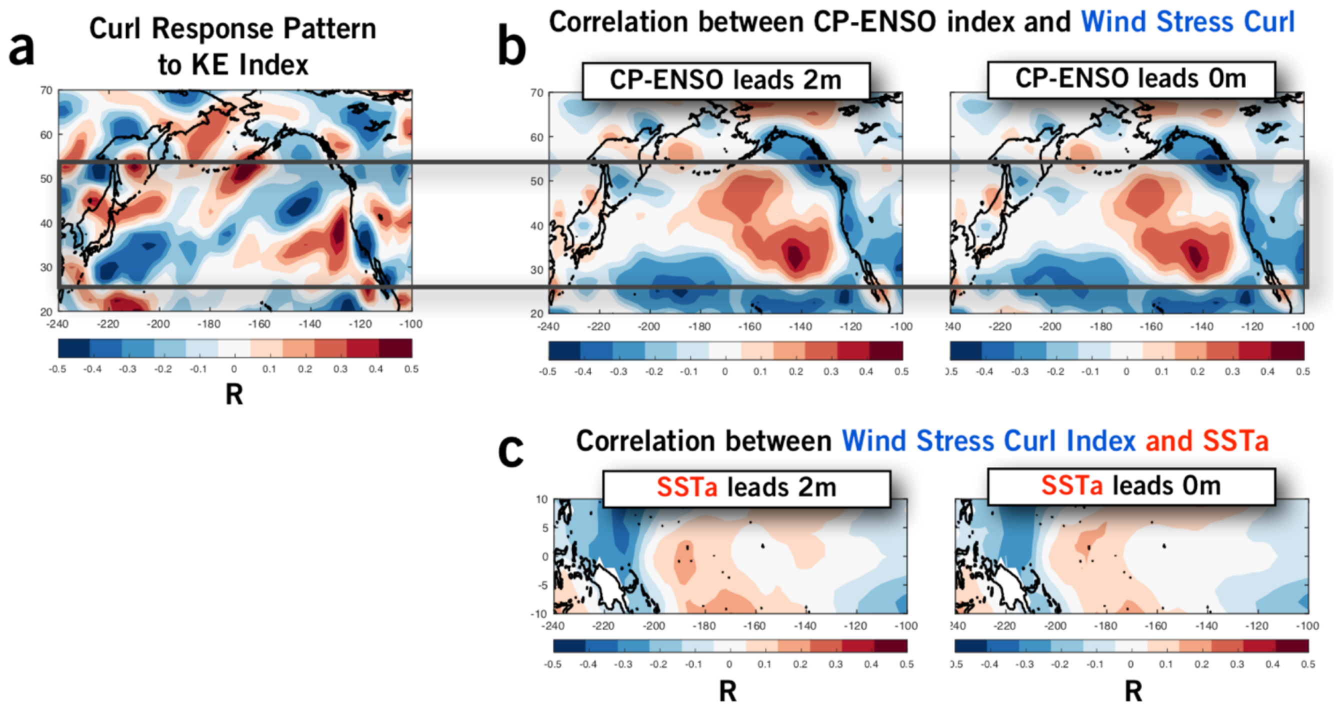

Figure S6. The role of the tropical Pacific in driving the KE wind stress curl response pattern. (a) The curl response pattern to the KE as shown in Figure 4a. (b) The curl response patterns to CP-ENSO anomalies extracted through a correlation of the CP-ENSO index with the wind stress curl anomalies at 2 and 0 months lead, whose structures show some important structural differences from the KE curl response pattern in panel a. (c) SSTa in the tropics impacting the KE curl response pattern extracted by correlating the KE curl index with SSTa at leads of 2 and 0 months. Weak correlations R=0.1-0.2 are found in the tropics indicating that some fraction of the variance in the KE curl response pattern may have tropical origin.

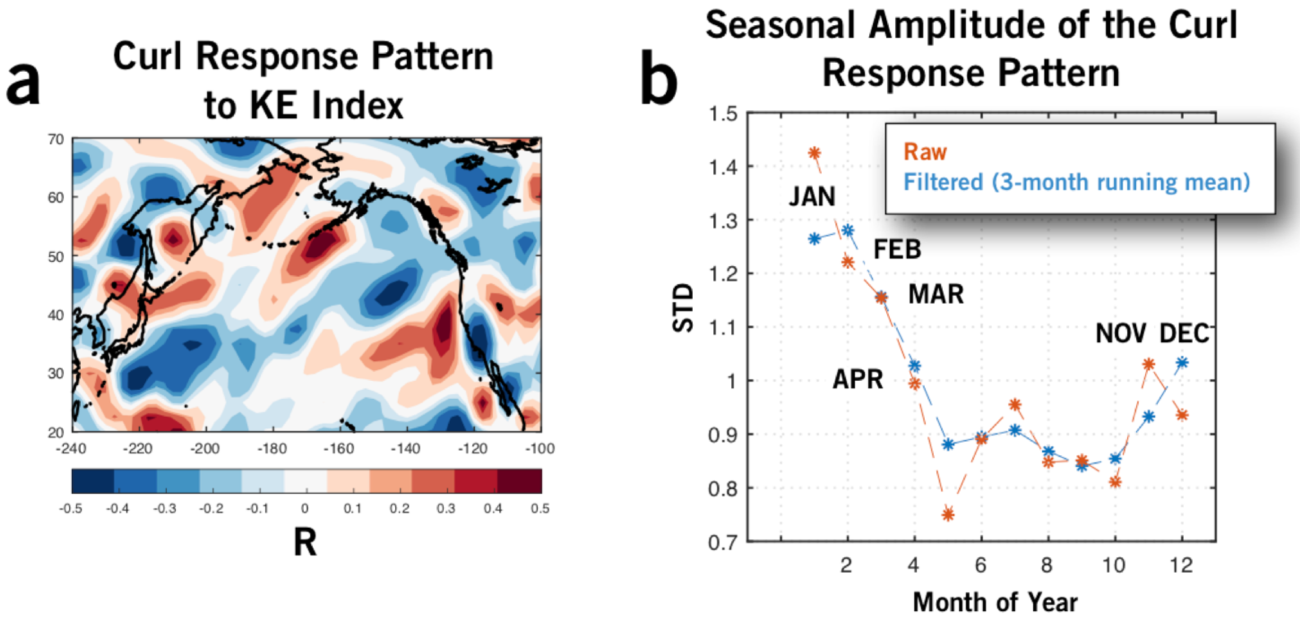

Figure S7. Seasonality of the KE wind stress curl response pattern. (a) The curl response pattern to the KE as shown in Figure 4a. (b) Seasonal standard deviation of the curl index extracted by projecting the KE curl pattern onto the raw and filtered (3-month running mean) monthly wind stress curl anomalies.
